# Evolving Insights Into the Biological Function and Clinical Significance of Long Noncoding RNA in Glioblastoma

**DOI:** 10.3389/fcell.2022.846864

**Published:** 2022-04-21

**Authors:** Kun Liu, Hong Chen, Yuanyuan Wang, Liping Jiang, Yi Li

**Affiliations:** ^1^ Department of Pathology, Northwestern University Feinberg School of Medicine, Chicago, IL, United States; ^2^ Department of Oncology, 920th Hospital of Joint Logistics Support Force, Teaching Hospital of Kunming Medical University, Kunming, China; ^3^ Department of Pathology, 920th Hospital of Joint Logistics Support Force, Teaching Hospital of Kunming Medical University, Kunming, China; ^4^ Key Laboratory of Animal Models and Human Disease Mechanisms of Chinese Academy of Sciences and Yunnan Province, Kunming Institute of Zoology, Kunming, China; ^5^ Department of Biochemistry and Molecular Genetics, University of Illinois at Chicago, Chicago, IL, United States

**Keywords:** lncRNA, glioblastoma, diagnostic biomarker, prognostic biomarker, targeted therapy

## Abstract

Glioblastoma (GBM) is one of the most prevalent and aggressive cancers worldwide. The overall survival period of GBM patients is only 15 months even with standard combination therapy. The absence of validated biomarkers for early diagnosis mainly accounts for worse clinical outcomes of GBM patients. Thus, there is an urgent requirement to characterize more biomarkers for the early diagnosis of GBM patients. In addition, the detailed molecular basis during GBM pathogenesis and oncogenesis is not fully understood, highlighting that it is of great significance to elucidate the molecular mechanisms of GBM initiation and development. Recently, accumulated pieces of evidence have revealed the central roles of long noncoding RNAs (lncRNAs) in the tumorigenesis and progression of GBM by binding with DNA, RNA, or protein. Targeting those oncogenic lncRNAs in GBM may be promising to develop more effective therapeutics. Furthermore, a better understanding of the biological function and underlying molecular basis of dysregulated lncRNAs in GBM initiation and development will offer new insights into GBM early diagnosis and develop novel treatments for GBM patients. Herein, this review builds on previous studies to summarize the dysregulated lncRNAs in GBM and their unique biological functions during GBM tumorigenesis and progression. In addition, new insights and challenges of lncRNA-based diagnostic and therapeutic potentials for GBM patients were also introduced.

## 1 Introduction

Glioblastoma (GBM) remains one of the most devastating and common malignancies of the central nervous system cancer in adults, and its worse prognosis has not achieved significant improvement despite the development of many new therapeutics (Brennan et al., 2013; Frattini et al., 2013). The median survival time of GBM patients remains only 15 months. Among the developed countries, an estimated more than 3.4 GBM incidents per 100,000 people are diagnosed each year (Ohgaki and Kleihues, 2005). Currently, the standard treatment of GBM is surgical resection followed by radiotherapy with concurrent temozolomide (TMZ) chemotherapy or adjuvant TMZ chemotherapy. A variety of new inhibitors specifically targeting diverse oncoproteins have been revealed to potently induce GBM regression (Shi et al., 2018; Shi et al., 2019a), but none of them has been approved for clinical applications for GBM therapy. To date, TMZ is still the only one approved by the FDA for GBM chemotherapy. Therefore, it is urgent for clinics to develop more agents for GBM. In addition, the failure of clinical treatments of GBM is attributed to the heterogeneity of GBM (Rusu et al., 2019; Qin et al., 2021) because GBM exhibits a high recurrence rate and frequent resistance to conventional therapeutics owing to tumor cells frequently invading into the surrounding brain, which renders surgical resection virtually impossible (Stupp et al., 2009; Oike et al., 2013). Conventional therapies, including radiotherapy and TMZ-dependent chemotherapy, only target a portion of proliferative cancer cells without harming other cancerous cells owing to the heterogeneity of GBM, which leads to frequent GBM relapse with poor prognosis. Therefore, there is an urgent need to decipher the uniquely biological principles of the initiation and progression of GBM and develop more novel biomarkers and reliable therapeutic targets to overcome this deadly disease.

Over the past several decades, numerous pieces of evidence reported that noncoding RNAs (ncRNAs) play central roles in GBM tumorigenesis and progression (Meller et al., 2015; Rutenberg-Schoenberg et al., 2016; Slack and Chinnaiyan, 2019). It has been well-accepted that the vast majority (more than 70%) of the genome is transcribed, whereas, no more than 2% of all the transcripts with coding potential and the remaining transcripts were originally considered to be transcriptional noise (Stackhouse et al., 2020). To date, speculation over the biological functions of ncRNAs has switched from transcriptional noise to master epigenetic regulators, and there is an increasing sense that genetic alterations in the noncoding region are the major causes of human disease, including malignant tumors (Maurano et al., 2012). Based on the current knowledge, ncRNAs are classified into two main categories depending on the size of transcripts. Long noncoding RNAs (LncRNAs) are defined as transcripts larger than 200 nucleotides (nt) with limiting or without protein-coding potential, including pseudogenes, lincRNAs, and circRNAs (Slack and Chinnaiyan, 2019; Chen et al., 2020a; Zhan et al., 2020). Notably, lncRNAs account for approximately 80–90% of ncRNAs. LncRNAs have attracted increasing attention for exerting fundamentally regulatory functions in various physiological and pathological processes (Rinn and Chang, 2012; Wahlestedt, 2013; Yin et al., 2015; Schmitt and Chang, 2016).

LncRNA was first identified in 1990 (Brannan et al., 1990a; Gabory et al., 2009). Subsequently, lncRNA XIST was identified to mediate X chromosome inactivation (Shi et al., 2020). At present, abundant genes have been documented to transcribe lncRNA, and this number is still increasing steadily and rapidly (Zhan et al., 2020). An important feature of lncRNAs is that lncRNAs are less conserved across different species and typically expressed lowly and frequently specifically expressed in a certain tissue or different developmental stages, which could be thought to be the most promising biomarkers for GBM diagnosis and prognosis (Gao et al., 2020a; Ghafouri-Fard et al., 2021). Functionally, LncRNA regulates gene expression *in cis* or *in trans* by physically interacting with different DNA, RNA, and proteins. Furthermore, with the progress of sequencing technology, the entire spectrum of lncRNAs is gradually investigated, suggesting that lncRNAs could function as not only effective indicators for the early diagnosis and determination of prognosis but also therapeutic targets for GBM. In this review, we introduced the molecular basis of lncRNA and comprehensively summarized the dysregulated lncRNAs involved in GBM tumorigenesis and development. In addition, We also introduced the clinical significance of diagnostic, prognostic, and therapeutic potential of lncRNAs in GBM.

## 2 Classification of lncRNA

**FIGURE 1:**
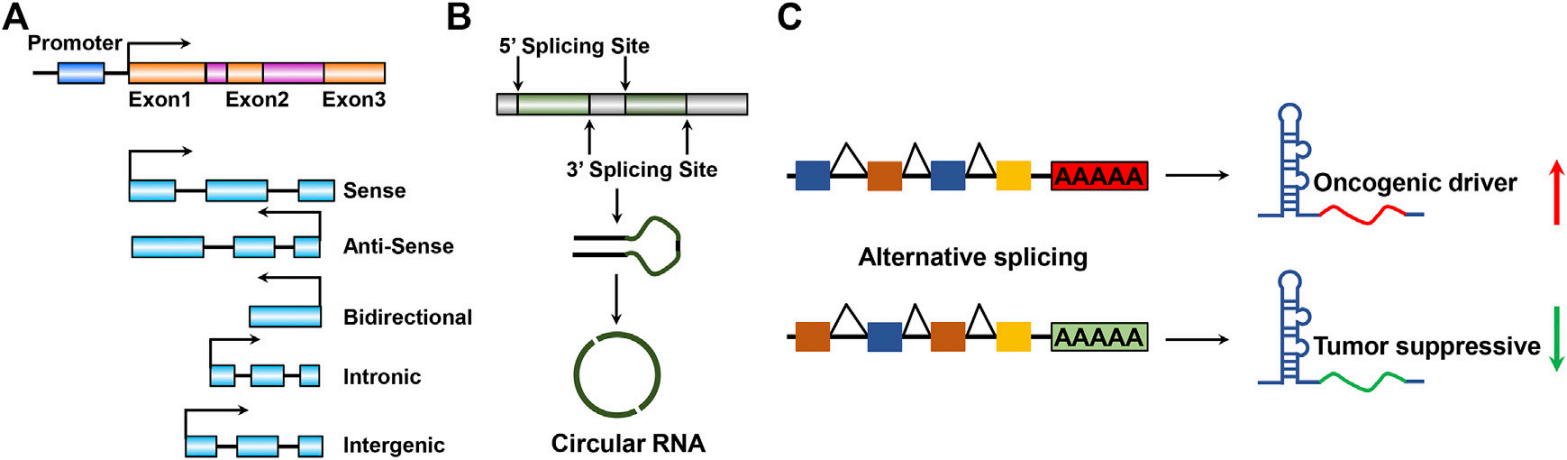
Schematic illustration of lncRNA species and structures. **(A)** Mainstream five categories according to the genomic location and transcript orientation of lncRNA. **(B)** Close circular structures in lncRNAs: originated from back-splicing of exons, namely, circular RNAs or circRNAs. **(C)**. LncRNA processing enables lncRNA by promoting or suppressing GBM progression properties during GBM tumorigenesis and development. Red and green arrows indicate highly or weakly expressed lncRNAs, respectively.

LncRNAs can be further divided into five categories based on the genomic location relevant to neighboring protein-coding genes (St. Laurent et al., 2015; Peng et al., 2018a): sense lncRNAs (Rinn and Chang, 2012), antisense lncRNAs (Ma et al., 2013), bidirectional lncRNAs (Stackhouse et al., 2020), intronic lncRNAs (Maurano et al., 2012), and intergenic lncRNAs (lincRNAs) (Figure 1A) (Ulitsky and Bartel, 2013). The order of nucleotide arrangement constitutes the primary structure of lncRNAs and the precise secondary and tertiary structures comprise the diversely biological functions of lncRNAs. Based on their subcellular localization (Quinn and Chang, 2016), lncRNAs can be further divided into nuclear or cytoplasmic lncRNAs (Chen, 2016). LncRNAs localized in the nucleus typically regulate gene expression *in cis,* including transcriptional interference or chromatin remodeling (Jain et al., 2016; Zhu et al., 2021). On the contrary, LncRNAs localized in the cytoplasm execute regulatory functions *via* implicating in influencing RNA processing or transport, thereby regulating mRNA degradation or directly altering protein function (Lin et al., 2016). Particular lncRNAs that are represented in a loop pattern, namely, circular RNAs (Meng et al., 2017) and circular intronic RNAs (circRNAs) (Zhang et al., 2013) originated from the back-splicing of exons or introns, respectively (Figure 1B). Notably, circRNAs attracted a great deal of research interest in regulating GBM pathogenesis and oncogenesis (Seimiya et al., 2020; Zhang et al., 2020; Nisar et al., 2021; Xu et al., 2021). Certain primary long noncoding transcripts process special processing, such as alternative splicing, which is an exon inclusion process for lncRNA genesis (Figure 1C). This special processing might offer a new oncogenic driver or tumor-suppressive function for GBM progression. The alternative splicing process events generate new lncRNAs involved in GBM pathogenesis and oncogenesis (Wang et al., 2021; Yang et al., 2021).

## 3 Generally Molecular Basis of lncRNAs

Since lncRNAs were discovered in 1990, numerous investigations have begun to determine how lncRNAs were involved in regulating signaling transduction in diverse cellular activity and multiple malignant tumor initiation and progression (Kopp and Mendell, 2018). LncRNAs exert their functional roles in regulating multiple biological events through various ways, including binding to DNA, interacting with RNA, and forming an RNA–protein complex through association with proteins (Figures 3, 4). First, chromatin structure and epigenetic modifications are remodeled by lncRNAs binding to DNA and thus affecting the expression of target genes. Second, lncRNAs function as molecular sponges by interacting with mRNAs or miRNAs, thereby mediating the stability and translation of mRNAs or the binding of miRNAs to affect their own target gene expression. Third, lncRNAs are able to bind with proteins to modulate their structural conformation, subcellular localization or stability, and other aspects of functions. We have discussed the functional roles of lncRNA in GBM in detail.

### 3.1 Transcriptional Activation

**FIGURE 2:**
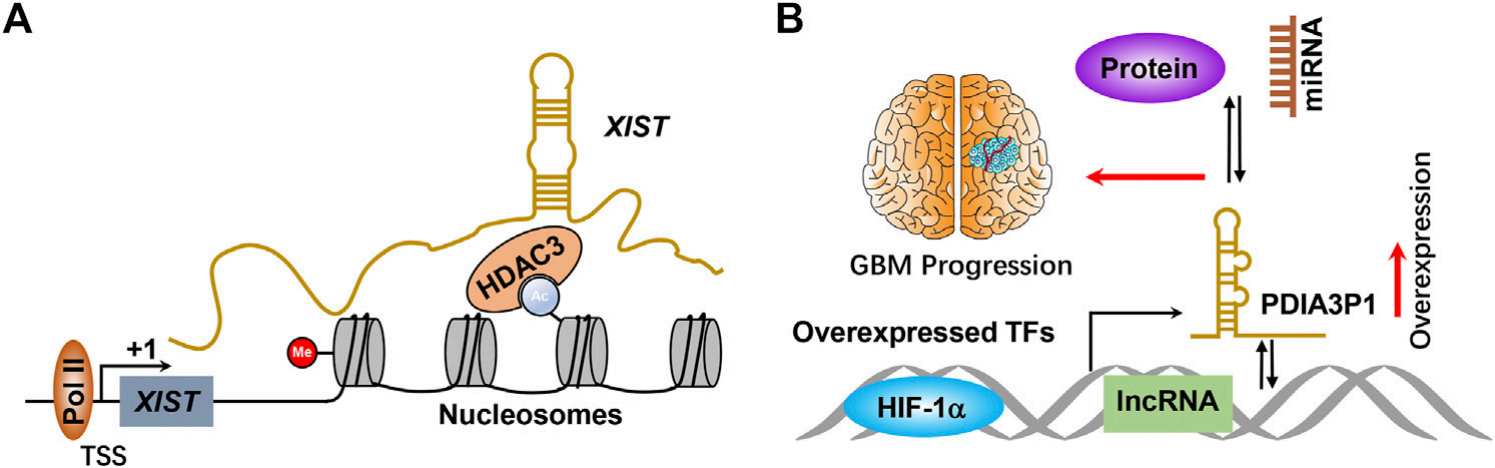
**(A)** Scheme of molecular mechanisms of lncRNA initiative transcriptional. TSS and +1 indicate transcriptional start sites and transcription begin, respectively. **(B)** HIF-1α or other transcription factors bind to the lncRNA promoter and activate lncRNA expression at the transcriptional level, thereby promoting GBM progression by binding with miRNA, protein, and DNA. TFs means transcription factors.

It has been widely accepted that many transcription factors are highly expressed in GBM, including SOX2, HIF-1α, and c-Myc (Zhang et al., 2011; Man et al., 2018; Zhou et al., 2016; Zheng et al., 2015; Bae et al., 2021). These transcription factors are not only involved in promoting the initiation of the transcriptional process of protein-coding genes but are also required for the initiative transcription of noncoding genes, including lncRNAs. XISTs were transcribed by general transcription factors involved in controlling gene expression by recruiting specific histone deacetylase to the nucleosomes (Figure 2A). For example, Wang et al. systemically reported that transcription factor HIF-1α was involved in transcribing numerous lncRNAs (Wang et al., 2020a; Liu et al., 2019a; Wang et al., 2020b). GBM is characterized by areas of hypoxia and is associated with worse patient survival. Thus, lncRNAs transcribed by HIF-1α may be partially responsible for HIF-1α–driven GBM progression. Wang et al. reported that PDIA3P1 was induced by HIF-1α and executed as ceRNA to sponge miR-124-3p, consequently promoting epithelial–mesenchymal transition in glioma (Figure 2B) (Wang et al., 2020a). Many lncRNAs have been revealed to be transcriptionally activated by transcription factors that play critical roles in promoting GBM progression, whereas the underlying molecular mechanisms of those lncRNAs in facilitating GBM progression require further study.

### 3.2 DNA Binding and Chromatin Remodeling

**FIGURE 3:**
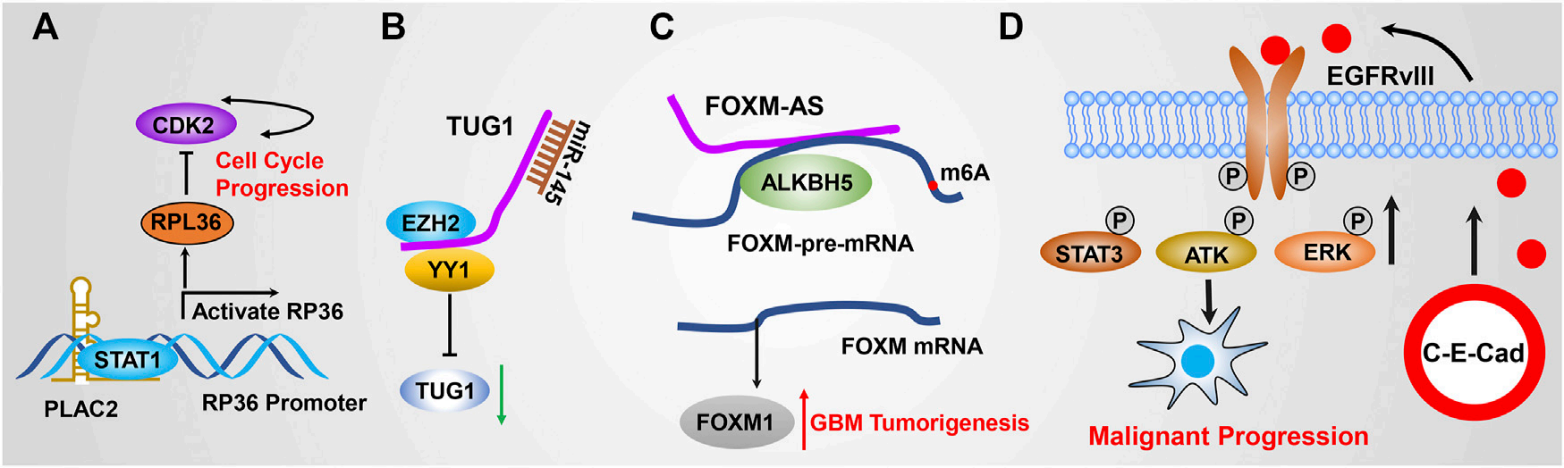
**(A)** PLAC2 interacts with STAT1 in the nucleus, thereby activating RPL36 expression in the transcriptional level through binding the RPL36 promoter, leading to inhibited GBM cell growth and arresting cell cycle progression. **(B)** TUG1 coordinately promotes GSC self-renewal by sponging miR-145 and recruiting EZH2 to repress differentiation gene expression. **(C)** FOXM1-AS promotes the interaction of ALKBH5 with FOXM1 nascent transcripts by specifically binding with ALKBH5. **(D)** Circular RNA E-cadherin encodes a small peptide that promotes GSC tumorigenicity through binding and activating EGFR signaling.

The vast majority of lncRNAs are found to localize in the nucleus, and are commonly found to bind with DNA to regulate gene expression. As mentioned previously, this direct RNA–DNA interaction renders lncRNAs to mediate gene expression *in cis* (lncRNA and target genes are localized at the same chromosome) or *in trans* (lncRNA and target genes are localized at different chromosomes); those lncRNAs commonly function as guide lncRNAs. The communication between lncRNAs and well-documented chromatin-associated regulatory complexes is recently further investigated. For example, the lncRNA SWINGN influences the capacity of the SWI/SNF complexes to trigger the epigenetic activation of specific promoters *via* SMARCB1-dependent activity in topologically organized regions (Grossi et al., 2020). XIST is one of the well-identified lncRNAs that specifically bind with DNA, which modulates X chromosome inactivation during the early developmental progression (Brown et al., 1991) (Figure 2A). To date, accumulated pieces of evidence have reported that multiple lncRNAs execute their biological function by binding with DNA. However, only a small fraction of lncRNA function has been fully elucidated. Shi et al. (2020) suggested that HOTAIRM1 represses the expression of HOXA cluster genes and GBM cell proliferation through the regulation of high-order chromatin structure. Hu et al. revealed that nucleus PLAC2 interacts with STAT1 and activates RPL36 in the transcriptional level by binding RPL36 promoters, but the cytoplasmic lncRNA PLAC2 repressed STAT1 nuclear transfer, consequently reducing RP36 expression, inhibiting glioma cell growth and arresting cell cycle progression (Figure 3A) (Hu et al., 2018a). It is noteworthy that only a small portion of lncRNAs was functionally illustrated to bind with DNA because of the lack of related studies.

### 3.3 miRNA Sponge

As mentioned previously, lncRNAs execute their biological function by interacting with various macromolecules to form RNA complexes. It has been widely studied that miRNA directly interacts with mRNAs to modulate mRNA stability, thereby regulating target gene expression (zur Hausen, 2008; Esquela-Kerscher, 2010). Thousands of lncRNAs have been identified to act as competitive endogenous RNA (ceRNA) by directly binding to miRNAs and affecting their stability; thus, these functional lncRNAs were also termed as miRNA sponges. For example, LncRNA SNHG4 directly binds to miR-138 and promotes GBM cell proliferation *via* upregulating c-Met, which is a target gene of miR-138 (Wang et al., 2020c). SNHG15 was highly expressed in GBM and promotes tumor angiogenesis by inhibiting the tumor suppressor gene miR-627-5p (Li et al., 2019a). GAPLINC promotes GBM cell proliferation and invasion by sponging miR-331-3p (Chen et al., 2019). LncRNA MALAT1 targets miR-199a and upregulates ZHX1 to promote glioma proliferation and progression (Liao et al., 2019a). Mu et al. suggested that BCYRN1 was the most highly expressed lncRNA in 183 dysregulated lncRNAs. BCYRN1 high expression predicts poor outcomes of glioma patients. BCYRN1 executed its function by sponging miR-619-5p, which was implicated in affecting CUEDC2 expression and the PTEN/AKT/p21 pathway (Mu et al., 2020). Katsushima et al. reported that TUG1 was specifically activated by Notch1 in GSCs coordinately potentiating GSC self-replication maintenance by sponging miR-145 in the cytoplasm and recruiting polycomb protein complexes to repress the expression of serval differentiation genes (Katsushima et al., 2016) (Figure 3B). Liu et al. (2021) reported that PCGEM1 inhibition repressed GBM cell proliferation, migration, invasion, and tumor growth *in vivo* by promoting CDK6 expression by quenching miR-539-5p. Deguchi et al. (2017) revealed that ECONEXIN as a novel oncogene promotes TOP2A expression by quenching miR-411-5p, which potentiates glioma cell proliferation. In addition, recent studies reported that LINC01094 (Liu et al., 2022), DGCR5 (He et al., 2020a), NFIA-AS2 (Xin et al., 2020), HANR (Wang et al., 2020d), LINC00355 (Qi et al., 2021), and ZFPM2-AS1 (Zhang et al., 2021) were involved in regulating GBM progression by quenching miR-224-5p, miR-21 and miR-23a, miR-655-3p, miRNA-335, miR-1225, and miR-515-5p, respectively. It is worth stating that numerous other functional lncRNAs, including HOTAIR, GAS5, and CASC2, are able to regulate GBM malignant progression by functioning as ceRNA to quench miRNA activity, which has also been reported elsewhere (Peng et al., 2018b; Cheng et al., 2020a; Stackhouse et al., 2020).

### 3.4 Protein Interaction

Multiple lncRNAs implement their regulatory functions by binding to RNA binding protein (RBP) and modulating their turnover, implying that lncRNAs hold great potential to serve as therapeutic targets. Given that various similarities existed between lncRNAs and mRNAs, many lncRNAs have been reported to be involved in regulating RBP stability by physical interaction with RBPs. LncRNA MATN1-AS1 binds to E2F6 by competing with RELA and decreased cell proliferation and migration. Kang et al. reported that lncRNA RP11 accelerates p21 degradation and promotes glioma growth by acting as a scaffold protein to physically interact with 14-3-3 β/α, which mediates the degradation of p21 (Kang et al., 2019). Miao *et al.* revealed that DLGAP1-AS2 promotes glioma cell proliferation, migration, and apoptosis *via* interacting and regulating YAP1 expression (Miao et al., 2020). Sheng et al. revealed that p53 directly binds to the promoter of ST7-AS1 and activates ST7-AS1 expression at the transcriptional level, which subsequently binds PTBP1 to suppress Wnt/β-catenin signaling and inhibits GBM progression (Sheng et al., 2021). Tang et al. revealed that LINC00115, which is activated by TGF-β, promotes GSC tumorigenicity by augmenting ZNF596 transcription *via* preventing binding of miR-200s to the 5′-UTR of ZNF596, thereby promoting ZNF596/EZH2/STAT3 signaling axis and GBM tumorigenesis (Yang et al., 2020a). Zhang et al. revealed that ALKBH5 demethylates FOXM1 nascent transcripts, resulting in increased FOXM1 expression. In addition, FOXM1-AS (a long non-coding RNA antisense to FOXM1) triggers the interaction of ALKBH5 with FOXM1 nascent transcripts by directly binding to ALKBH5. Silencing ALKBH5 and FOXM1-AS blocked GSC tumorigenesis by the FOXM1 axis (Zhang et al., 2017a) (Figure 3C). These pieces of evidence suggest that lncRNAs could be involved in signaling transduction by regulating RBP stability, activation, or subcellular localization.

LncRNA was also involved in regulating protein posttranscriptional modification by directly interacting with protein. For instance, Zhu et al. revealed that lnc-β-Catm functions as a scaffold lncRNA to interact with β-catenin and the methyltransferase EZH2, resulting in methylating β-catenin. Methylation of β-catenin inhibits the ubiquitination and β-catenin protein accumulation, thereby activating Wnt-β-catenin signaling and liver CSC self-renewal (Zhu et al., 2016a). Wang et al. suggested that lnc-DC (dendritic cells) interacts directly with STAT3 in the cytoplasm, which leads to enhanced STAT3 phosphorylation on tyrosine-705 *via* preventing STAT3 binding to and dephosphorylation by SHP1 (Wang et al., 2014). Another study suggested that HIF-1α–induced lincRNA-p21 in the transcriptional level and highly expressed lincRNA-p21 associates with HIF-1α and VHL, thereby leading to disrupting the VHL/HIF-1α protein complex interaction and resulting in attenuating VHL-mediated HIF-1α ubiquitination and degradation, which is essential for hypoxia-mediated glycolysis and cancer progression (Yang et al., 2014). This study highlighted that lncRNA-p21 serves as a valuable therapeutic target for cancer. Furthermore, Sun et al. revealed that lncRNA GClnc1 activates gastric carcinogenesis progression by serving as a modular scaffold of WDR5, which is a key component of histone methyltransferase complex and KAT2A, which consequently leads to altered histone modification pattern (Sun et al., 2016).

As introduced previously, lncRNA is able to serve as an anchoring scaffold by directly binding to the protein complex. For instance, Bian et al. reported that FEZF1-AS1 is capable of binding and enhancing pyruvate kinase 2 (PKM2) protein stability, which causes improved cytoplasmic and nuclear PKM2 levels. Increased cytoplasmic PKM2 boosted pyruvate kinase activity and lactate production, thereby leading to promoting colorectal cancer progression (Bian et al., 2018). However, to the best of our knowledge, there are no GBM-associated lncRNAs involved in protein posttranscriptional regulation. Therefore, it is necessary to explore GBM-associated lncRNAs involved in promoting key protein stability.

### 3.5 Encoding Small Functional Micro-Peptides

As mentioned above, lncRNAs are defined with transcripts longer than 200 nt and without coding capability. However, much evidence supported that lncRNAs execute their function by encoding functional micropeptides based on their small open-reading frames (sORFs) (Anderson et al., 2015). As early as 2002, lncRNA ENOD40 was able to encode a small peptide, which directly interacts with sucrose synthase and thereby affects its biological function (Rohrig et al., 2002). Following the discovery of bioinformatic approaches and next-generation sequencing technologies in recent years, more and more sORFs have been characterized in transcripts formerly recognized as noncoding potential (Ingolia et al., 2014; Aspden et al., 2014; Huang et al., 2020a). For example, circular RNA E-cadherin, which is encoded by the E-cadherin variant, promotes GSC tumorigenicity by encoding a small peptide called C-E-Cad, which then binds to EGFR, thereby activating EGFR/STAT3 signaling. Targeting C-E-Cad by an antibody against C-E-Cad inhibits GSC tumorigenic capacity *in vivo* (Gao et al., 2021) (Figure 3D). Zhang et al. revealed that an 87–amino-acid small peptide encoded by LINC-PINT regresses GBM cell proliferation *in vitro* and *in vivo* by directly interacting with PAF1c and thereby suppressing the transcriptional elongation of various oncogenes (Zhang et al., 2018). These landmark studies open a new direction for the GBM studies and provide more options for developing new therapeutic targets for GBM patients.

### 3.6 Exosome-Transmitted lncRNA

Exosomes (70–120 nm) are microvesicles that are derived from multivesicular bodies (MVBs) and are secreted into the extracellular milieu after fusion with the cytomembrane (Théry, 2011). Recently, emerging pieces of evidence demonstrated that exosomes can be released from various types of cells and can participate in intercellular communication by transmitting intracellular regulators, such as proteins and nucleic acids, including miRNA and lncRNA (Peinado et al., 2012; Melo et al., 2014). A landmark study from Qu et al. revealed that exosomal lncARSR derived from resistant renal cell carcinoma (RCC) cells could confer sunitinib resistance to endothelial cells by functioning as a ceRNA to quench miR-34 and miR-449, thereby leading to accumulated AXL and c-MET expression. Targeting lncARSR or AXL/c-MET in sunitinib-resistant RCC restores drug sensitivity (Qu et al., 2016). In GBM, exosomes derived from AHIF (Dai et al., 2019), LINC00470 (Ma et al., 2021), and lncSBF2-AS1 reportedly facilitate GBM malignant progression.

## 4 Significance of lncRNAs in GBM Malignant Phenotypes

Recently, numerous studies documented that multiple dysregulated lncRNAs in GBM are closely associated with GBM tumorigenesis and malignant progression (Yan et al., 2017; Pan et al., 2020; Li et al., 2021). GBM-related lncRNAs that facilitate or repress these malignant behaviors by interplaying with their binding partners are comprehensively summarized as below.

### 4.1 Gaining GSC Properties

**FIGURE 4:**
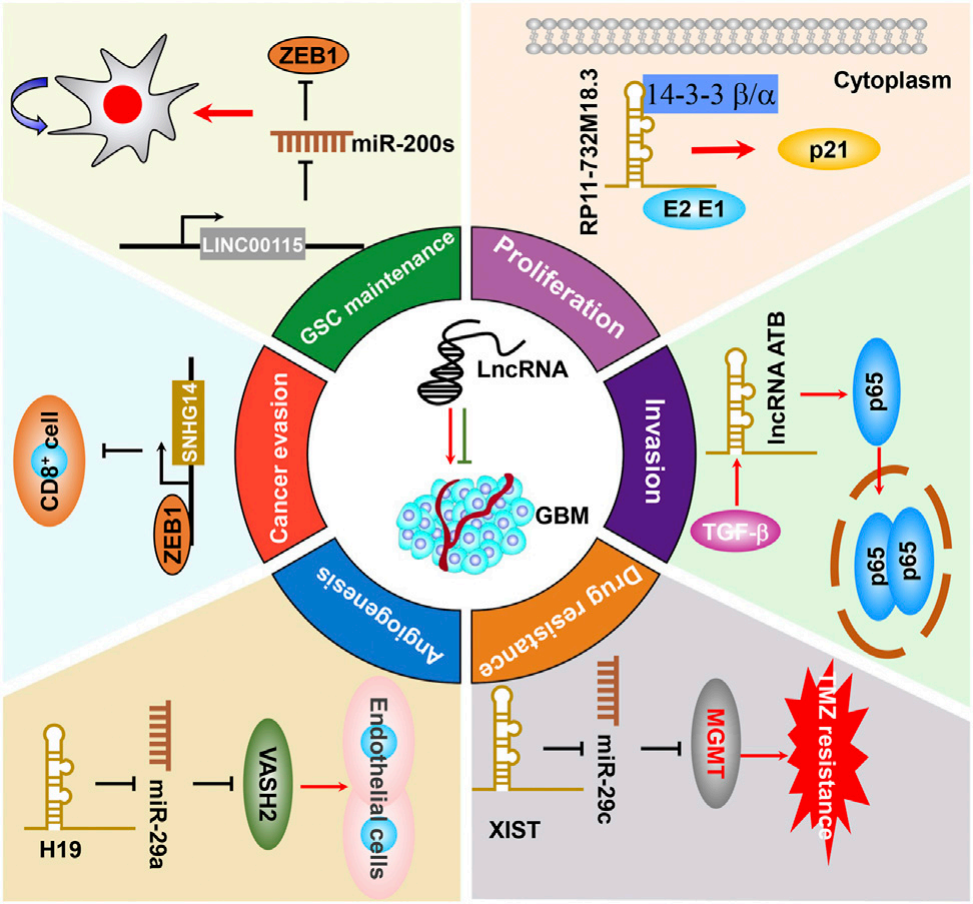
The functional roles and molecular mechanisms of lncRNAs in sustaining or suppressing GBM malignant progression.

It has been well-proven that a small subset of cancerous cells, termed as the glioma stem cell (GSC) in glioma (Hemmati et al., 2003; Singh et al., 2003; Singh et al., 2004), bear extremely high tumorigenic potential by replicating rapidly and differentiating into specialized cell types. GSCs have been shown to account for GBM malignant progression, recurrence, and therapeutic resistance (Bao et al., 2006; Chen et al., 2012; Cai et al., 2021). At present, emerging pieces of evidence revealed that lncRNAs were also well-established to be crucially involved in regulating GSC self-renewal maintenance. For example, LINC00115, activated by TGF-β, acts as a miRNA sponge and upregulates ZEB1 by competitively binding to miR-200s, thereby increasing ZEB1 signaling and GSC self-renewal ability (Figure 4) (Liu et al., 2019b). In addition, MALAT1 was reported to be enriched in GSC extracellular vesicles compared with GSCs and directly interacts with miR-129-5p, thereby decreasing HMGB1 expression (Yang et al., 2020a). In another study, MALAT1 was reported to promote GSC self-renewal and proliferation by increasing SOX2 expression, which is an important GSC stemness maintenance regulator (Ke et al., 2015). As mentioned above, TUG1 was specifically triggered by Notch1 in to coordinately promote GSC self-renewal by sponging miR-145 in the cytoplasm and recruiting polycomb protein complexes to suppress serval differentiation gene expression (Katsushima et al., 2016). LncRNA XIST was found to potentiate the proliferation of GSCs and regressed their apoptotic rate. More than directly sponging to miR-152, XIST is likely in the same RNA-induced silencing complex (RISC) with miR-152, leading to abrogate miR-152 downstream signaling (Yao et al., 2015). All of these findings suggest that lncRNAs could function as an important regulator in regulating GSC self-renewal and hold tremendous potential to serve as therapeutic targets to specifically eliminate GSCs. Thus, it is of significance to decipher more oncogenic lncRNAs and their molecular basis that promote GSC self-renewal.

### 4.2 Promoting Uncontrolled Proliferation

Glioma tumorigenesis is a biologically complex process consisting of abnormal activation of oncogenic drivers or loss of function of tumor suppressors (Hanahan and Weinberg, 2000; Chinigò et al., 2021). As mentioned previously, lncRNAs play important roles in regulating the malignant proliferation of GBM or other cancers. For instance, the expression of lncRNA CCND2-AS2 is significantly increased in GBM tissues and cell lines. Targeting CCND2-AS1 represses GBM cell growth and migration through suppressing Wnt/β-catenin signaling (Zhang et al., 2017b). LncRNA NNT-AS1 potentiates glioma cell proliferation *via* quenching miR-494-3p, resulting in upregulating PRMT1 and promoting GBM progression (Zheng et al., 2020a). As mentioned previously, Deguchi et al. (2017) reported that ECONEXIN is a novel oncogene that promotes TOP2A expression through quenching miR-411-5p, which potentiates glioma cell proliferation. Kang et al. (2019) reported that lncRNA RP11-732M18.3 specifically binds 14-3-3 β/α, which facilitates p21 degradation, thereby promoting GBM cell proliferation (Figure 4). It is worth mentioning that several lncRNAs, including MATN1-AS1 (Zhu et al., 2020), ROR1-AS1 (Chai et al., 2020), and LEF1-AS1 (Cheng et al., 2020b), are capable of influencing GBM uncontrolled cell proliferation through binding with other molecules, which have been summarized elsewhere (Li et al., 2018). In addition, the dysregulated lncRNAs that regulate GBM cell proliferation are listed in detail in Table 1.

**TABLE 1:** Dysregulated lncRNAs and their biological significance in the progression of GBM.

LncRNAs	Roles	Partners	Working models	Significance	References
LINC00115	Oncogene	miR-200s	miRNA sponge	Self-renewal	Liu et al. (2019b)
MALAT1	Oncogene	miR-129-5p	miRNA sponge	Self-renewal	Han et al., 2016; Yang et al., 2020a
TUG1	Oncogene	miR-145	miRNA sponge	Self-renewal	Katsushima et al. (2016)
CGEM1	Oncogene	miR-539-5p	miRNA sponge	Proliferation; migration	Liu et al. (2021)
DGCR5	Tumor suppressor	miR-21, miR-23a	miRNA sponge	Invasion	He et al. (2020a)
NFIA-AS2	Oncogene	miR-655-3p	miRNA sponge	Proliferation; migration	Xin et al. (2020)
HANR	Oncogene	miRNA-335	miRNA sponge	Proliferation; invasion	Wang et al. (2020d)
LINC00355	Oncogene	miR-1225	miRNA sponge	Proliferation; invasion	Qi et al. (2021)
HOTAIR	Oncogene	miR-326	miRNA sponge	Proliferation; invasion	Ke et al. (2015)
GAS5	Tumor suppressor	miR-196a-5p	miRNA sponge	Proliferation; invasion	Zhao et al. (2017)
XIST	Oncogene	miR-152	miRNA sponge	Self-renewal; drug resistance	Yao et al., 2015; Du et al., 2017b
HOTAIRM1	Oncogene	HOX	Transcriptional activation	Self-renewal	Xia et al. (2020)
LINC00115	Oncogene	miR-200s	miRNA sponge	Self-renewal	Tang et al. (2019b)
CCND2-AS2	Oncogene	Wnt/β-catenin	Protein degradation	Proliferation; migration	Zhang et al. (2017b)
NNT-AS1	Oncogene	miR-494-3p	miRNA sponge	Proliferation	Zheng et al. (2020a)
ECONEXIN	Oncogene	miR-411-5p	miRNA sponge	Proliferation	Deguchi et al. (2017)
RP11-732M18.3	Oncogene	14-3-3 β/α	Protein degradation	Proliferation	Kang et al. (2019)
SNHG1	Oncogene	miR-194, miR-9-5p	miRNA sponge	Proliferation; migration	Liu et al. (2019b); Mi et al. (2020)
BLACAT1	Oncogene	miR-605-3p	miRNA sponge	Proliferation; migration	Liu et al. (2019c)
GACAT3	Oncogene	miR-135a	miRNA sponge	Proliferation; migration	Wang et al. (2019b)
LINC00475	Tumor suppressor	miR-449b-5p	miRNA sponge	Proliferation; migration	Yu et al. (2020)
MATN1-AS1	Oncogene	miR-200b/c/429	miRNA sponge	Proliferation	Zhu et al. (2020)
ROR1-AS1	Oncogene	miR-4686	miRNA sponge	Proliferation	Chai et al. (2020)
LEF1-AS1	Oncogene	miR-489-3p	miRNA sponge	Proliferation	Cheng et al. (2020b)
LINC00470	Oncogene	miR-580-3p	miRNA sponge	Proliferation	Ma et al. (2021)
FOXD1-AS1	Oncogene	miR339-5p	miRNA sponge	Proliferation; migration	Gao et al. (2020b)
AC016405.3	Tumor suppressor	miR-19a-5p	miRNA sponge	Proliferation; migration	Ren and Xu, (2019)
NCK1-AS1	Oncogene	miR-138-2-3p	miRNA sponge	Proliferation; migration	Huang et al. (2020b)
MIR22HG	Oncogene	miR-22	miRNA sponge	Proliferation; self-renewal	Han et al. (2020b)
BCAR4	Oncogene	EGFR	Protein binding	Proliferation	Wei et al. (2019)
THOR	Oncogene	IGF2BP1	Protein binding	Proliferation	Xue et al. (2019)
PDIA3P1	Oncogene	miR-124-3p	miRNA sponge	Invasion	Wang et al. (2020a)
ATB	Oncogene	p38	Protein binding	Invasion	Tang et al. (2019a)
PVT1	Oncogene	miR-128-3p	miRNA sponge	Invasion	Fu et al. (2018)
NEAT1	Oncogene	miR-132	miRNA sponge	Proliferation, invasion	Zhou et al., 2018; Zhen et al., 2019
RMST	Oncogene	FUS	Protein degradation	Proliferation; invasion	Liu et al. (2020c)
PAXIP1-AS1	Oncogene	ETS1	Transcriptional activation	Invasion; angiogenesis	Xu et al. (2019)
SOX2OT	Oncogene	ALKBH5	Protein scaffold	Drug resistance	Liu et al. (2020a)
CASC2	Oncogene	miR-181a	miRNA sponge	Drug resistance	Liao et al. (2017a)
TP73-AS1	Oncogene	ALDH1A1	Transcriptional activation	Drug resistance	Mazor et al. (2019)
SNHG15	Oncogene	miR-627-5p	miRNA sponge	Drug resistance	Li et al. (2019b)
SBF2-AS1	Oncogene	miR-151a-3p	miRNA sponge	Drug resistance	Zhang et al. (2019)
ADAMTS9-AS2	Oncogene	FUS	Protein binding	Drug resistance	Yan et al. (2019)
CASC2	Oncogene	miR-181a	miRNA sponge	Drug resistance	Liao et al. (2017b)
H19	Oncogene	miR-29a	miRNA sponge	Angiogenesis	Jia et al. (2016)
HULC	Oncogene	PI3K/Akt/mTOR	Protein scaffold	Angiogenesis	Zhu et al. (2016b)
NKILAT	Oncogene	HIF-1α	Not reported	Angiogenesis	Chen et al. (2020b)
LINC00346	Oncogene	ZNF665	mRNA binding	Angiogenesis	Yang et al. (2020b)
LINC00667	Oncogene	miR-429	mRNA binding	Angiogenesis	Wang et al. (2019a)
LINC-RA1	Oncogene	H2Bub1/USP44	Protein scaffold	Radioresistance	Zheng et al. (2020b)

Avoiding cellular apoptosis is another avenue for cancer cells to promote cell proliferation. Recent studies suggested that lncRNAs also play critical roles in cellular apoptosis (Liu and Gu, 2022; Misir et al., 2022; Roberts et al., 2022). For instance, Cheng et al. underscored that STAT1 enhances NKILA expression through binding with the NKILA promoter and consequently transcriptionally activates NKILA expression, which results in regulating T cell sensitivity to activation-induced cell death by inhibiting NF-κB activity by interacting with NF-κB. High expression of NKILA in tumor-specific cytotoxic T lymphocytes and T_H_1 cells is associated with their apoptosis and shorter patient survival (Huang et al., 2018). However, the functional roles of lncRNAs in GBM cell death, including autophagy, pyroptosis, and ferroptosis, still remain to be understood.

### 4.3 Activating Invasion and Metastasis

Tumor metastasis remains the leading cause of mortality in cancer patients worldwide. Generally, metastasis is a complicated biological process with numerous stochastic events, including cancer cell migration, local invasion, cancer cell intravasation into the circulation, seed at secondary sites, and formation of clinically detectable metastasis (Lambert et al., 2017; Massagué and Obenauf, 2016). Compelling evidence demonstrated that lncRNAs played critical roles in regulating GBM metastatic progression. For example, lncRNA PDIA3P1, which was activated by hypoxia, and its high expression was correlated with the process of epithelial–mesenchymal transition (EMT) and angiogenesis, and ectopically expressed PDIA3P1 potentiates the migration and invasion capacity of GBM cells (Wang et al., 2020a). Feng et al. recognized that lncRNA-ATB, which is activated by TGF-β, augmented glioma cell invasion mediated by TGF-β through activating p38 signaling (Figure 4) (Tang et al., 2019a). Fu et al. reported that lncRNA PVT1 functions as a ceRNA sponge of miR-128-3p and enhances GBM cell proliferation and invasion by affecting BMP2 and BMP4 expression, which are the core regulators of the BMP signaling pathway (Fu et al., 2018). Zhou et al. recognized that NEAT1 expression was significantly increased in glioma. Targeting NEAT1 regressed glioma cell proliferation, migration, and invasion through sponging miR-132, thereby inhibiting Sox2 expression (Zhou et al., 2018). All of these studies not only provide significant insights into the metastatic progression of biological functions of lncRNAs but also identify new targeted biomarkers, which can be utilized in the targeted therapy for GBM clinical treatment.

EMT is an intricate complex that is required for the invasion of GBM cells and gives rise to a new tumor (Dong et al., 2021). However, the biological function of lncRNAs in the EMT process of GBM remains poorly understood. Recently, Tao et al. screened EMT-associated lncRNAs in TCGA database and found nine EMT-associated lncRNAs and GBM patients with higher EMT-associated lncRNA expression had poorer overall survival (Tao et al., 2021). However, the biological function and underlying molecular basis of EMT-associated lncRNAs still need to be characterized.

### 4.4 Therapeutic Resistance

Currently, the primary therapy for GBM is TMZ-based chemotherapy or radiotherapy and surgery (Ethun et al., 2017; Han et al., 2020a). As mentioned previously, TMZ is routinely used in glioma patient chemotherapy (Burster et al., 2021). However, the significant challenge of GBM treatments is that the patients eventually develop chemoresistance to TMZ, which consequently causes tumor recurrence. LncRNAs are also involved in TMZ resistance. For instance, lncRNA SOX2OT recruits ALKBH5 to demethylate the SOX2 transcript, thereby leading to increased SOX2 expression, which then promotes GBM cell resistance TMZ treatment (Liu et al., 2020a). Besides the promotion of GBM cell growth and migration, the lncRNA MALAT1 was also documented to be resistant to TMZ in GBM, and silencing MALAT1 sensitizes GBM to TMZ treatment *in vitro* (Li et al., 2017; Kim et al., 2018). DNA repair protein O-6-methylguanine-DNA methyltransferase (MGMT) plays an important role in TMZ resistance. XIST potentiates the chemoresistance of GBM cells to TMZ by directly quenching and inhibiting miR-29c expression, which then targets SP1 and MGMT and decreases SP1 and MGMT expression. Thus, XIST/miR-29c may be a potential therapeutic target for glioma treatment (Figure 4) (Du et al., 2017a). LncRNA CASC2 was weakly expressed in GBM, and exogenous CACS2 alone inhibited GBM cell proliferation and amplified TMZ-induced apoptosis of cell proliferation (Liao et al., 2017a). TP73-AS1 amplifies TMZ resistance in GSC and activates metabolism-related gene expression. ALDH1A1 is a biomarker known to be primarily expressed in GSCs and protects GSC from TMZ treatment (Mazor et al., 2019). Silencing SNHG15 in TMZ resistant cells enables GBM cells to be significantly sensitive to TMZ therapy by inhibiting miR-627-5p expression, which results in activation of the CDK6 (Li et al., 2019b). In addition, another report revealed that combining p50 and p53 with the proximal κB and p53 sites of the MALAT1 coding region, thereby increasing the chemosensitivity in turn (Voce et al., 2019). LncSBF2-AS1 was found to be highly expressed in TMZ-resistant GBM cells and tissues, targeting lncSBF2-AS1–sensitized resistant GBM cells to TMZ resistance through serving as ceRNA for sponging miR-151a-3p, resulting in the suppression of its endogenous target, X-ray repair cross-complementing 4 (XRCC4), which increases DNA double-strand break repairability in GBM cells (Zhang et al., 2019). Higher expression of ADAMTS9-AS2 correlated with worse TMZ therapeutic effects and shorter progression-free survival (PFS) in TMZ-treated GBM patients. Silencing of ADAMTS9-AS2 prohibited GBM proliferation, migration, and invasion and decreased the therapeutic effects of TMZ treatment by directly binding to the RRM and Znf_RanBP2 domains of FUS, leading to increased FUS protein expression (Yan et al., 2019). In addition, the involvement of lncRNAs in TMZ resistance is listed in Table 1. As a consequence, it is feasible in clinics to specifically target lncRNAs to offer a fundamental option to overcome TMZ resistance in GBM. LncRNA is involved in TMZ.

Radiotherapy was considered to be the standard therapy for GBM patients. However, its therapeutic benefits are frequently limited with the development of radiotherapy resistance. LncRNAs emerged as regulators in radioresistance (Cardon et al., 2021). Our previous study suggested that high expression of KCNQ1OT1 was observed in stereotactic body radiotherapy-resistant cells and tissues, positively correlated with advanced clinical stage, and lower response rate to concurrent therapy. Silencing KCNQ1OT1 resensitized A549/IR and H1975/IR cells to radiation by prohibiting autophagy through sponging miR-372-3p, which directly targets autophagy-related 5 (ATG5) and autophagy-related 12 (ATG12) (He et al., 2020b). Liao et al. suggested that the antisense transcript of hypoxia-inducible factor-1α (AHIF) was highly expressed in GBM cells upon radiotherapy. Targeting AHIF repressed GBM cell clonogenic formation, DNA repair ability, and induced cellular apoptosis. Notably, knockdown of AHIF inhibited tumorigenesis after radiotherapy *in vivo* (Liao et al., 2019b).

### 4.5 Abnormal Angiogenesis

Solid tumors enhanced the requirement for oxygen and nutrient exchange owing to the rapid growth of cancer cells. Thus, abnormal angiogenesis was extremely required for cancer cells for proliferation and metastasis. Tumor cells are capable of promoting angiogenesis or even differentiating into endothelial cells to form new vessels (Pezzella et al., 2015; Wang et al., 2020b). Recent advances reported that many lncRNAs are involved in mediating the abnormal angiogenesis of GBM (Balandeh et al., 2021). For instance, Jia et al. (2016) characterized that H19 promotes glioma-associated endothelial cell (GEC) growth and the formation of the tube through sponging miR-29a, thereby promoting angiogenic factor VASH2 expression and angiogenesis. Yu et al. (2017) demonstrated that XIST promotes GBM angiogenesis *via* activation of the transcriptional activity of chemokine receptor 7b (CXCR7). The following study further proved that XIST potentiates glioma angiogenesis by sponging miR-429 (Cheng et al., 2017). LncRNA HULC (Zhu et al., 2016b) and H19 (Liu et al., 2020b) were identified to promote angiogenesis by activating the PI3K/Akt/mTOR and prohibiting the HIF-1α signaling pathway, respectively. Chen et al. elucidated that ectopic expression of NKILAT was inversely associated with the overall survival of GBM patients and high NKILAT expression potentiates angiogenesis in glioma, implying that NKILAT holds the potential to serve as a promising therapeutic biomarker by simulating HIF-1α signaling (Chen et al., 2020b). Likewise, Wang et al. reported that targeting USF1 potently repressed angiogenesis in GBM *via* SNHG16/miR-212-3p and the LINC00667/miR-429 signaling axis (Wang et al., 2019a).

### 4.6 Immune Evasion

The tumor immune microenvironment is closely associated with the aggressive development and therapeutic resistance of GBM. GBM cells commonly exhibit immune evasion or even interact with immune cells, thereby triggering malignant progression by secreting cytokines (Lei and Lee, 2021; Corral-Jara et al., 2021). LncRNAs were also involved in mediating immune evasion in GBM progression. For instance, Wang et al. (2020e) identified five immune gene–related lncRNAs (AP001007.1, LBX-AS1, MIR155HG, MAPT-AS1, and LINC00515) that were correlated to glioma patient prognosis and clinical characteristics and are positively correlated with PD-L1, TIM-3, and B7-H3 expression. Zhao et al. (2019) reported that ZEB1 transcriptionally activated SNHG14, which displays immune evasion effects by inhibiting cytotoxic cell activation (Figure 4). ROR1-AS1 was found to be packaged into exosomes and derived from tumor cells. Functional analysis suggested that exo-ROR1-AS1 facilitates GBM development through sponging miR-4686 (Chai et al., 2020). However, there are limiting pieces of evidence demonstrating the functional roles of lncRNAs in the progression of GBM immune evasion, suggesting that there is a crying need to identify more GBM immune evasion–associated lncRNA and decode their biological function.

Many studies have shown that cancerous cells are capable of using epigenetic mechanisms to alter their autoimmune origin and destroy the recognition process between tumor cells and the immune system by DNA methylation and histone modifications, which decrease the expression of critical molecules in the immunoreaction process, thereby resulting in disrupting the immune recognition and the failure of immunotherapy (Zang et al., 2018). Currently, many epigenetic inhibitors, including histone deacetylase inhibitors (HDACI), vorinostat (Lee et al., 2012) and valproic acid (Su et al., 2011), or DNA methyltransferase (DNMT) inhibitors, 5-aza-20-deoxycytidine (Garcia-Manero et al., 2006), have entered clinical trials either individually or in combination with TMZ for GBM. Thus, the combination of epigenetic drugs and tumor immunotherapy is a promising trend for developing cancer therapy. LncRNAs are critical epigenetic regulators with critical roles in cancer widely participating in epigenetic modification. For instance, Pastori et al. used Helicos single-molecule sequencing to comprehensively profile differentially expressed lncRNAs in GBM, and a subset of GBM-specific lncRNAs regulated by bromodomain and extraterminal (BET) proteins were identified. Treatment of GBM cells with the BET bromodomain inhibitor I-BET151 attenuated oncogenic lncRNA HOTAIR and revitalized a variety of other GBMs decreased lncRNA expression (Pastori et al., 2015).

## 5 Clinical Potential of lncRNAs in GBM

To date, surgical resection is the only likely curative strategy for low-grade glioma, while GBM in the late stage is largely incurable. Thus, new biomarkers and therapeutic targets are urgently required for advancing the diagnosis and clinical therapy of GBM. Here, we summarized the dysregulated lncRNAs in GBM and introduced the diagnostic and prognostic potential of lncRNAs in GBM.

### 5.1 Aberrant Expression of lncRNAs in GBM

Ever since the discovery of lncRNA in fetal liver tissue in 1990 (Brannan et al., 1990b), aberrant lncRNA biogenesis has been reportedly observed in the pathogenesis of a variety of diseases, including GBM. In GBM, Mu et al. implemented high-throughput RNA sequencing in three fresh GBM patient specimens and three normal brain tissues from craniocerebral trauma patients and identified that 183 lncRNAs were significantly dysregulated in glioma (Mu et al., 2020). Among the highly expressed lncRNAs, BCYRN1 was recognized to be the most highly expressed in GBM, and further functional studies demonstrated that BCYRN1 significantly promotes GBM cell proliferation and migration (Mu et al., 2020). Li et al. characterized 247 immune-correlated lncRNAs from 529 low-grade glioma samples and five nontumor brain tissue samples in TCGA public database. Cox regression analysis demonstrated that 16 immune-related lncRNA expressions are correlated with the prognosis in low-grade glioma patients (Li and Meng, 2019). As shown in Table 1, we summarized the dysregulated lncRNAs in glioma and their unique biological significance in glioma occurrence and malignant development. Given the relatively easier examination of lncRNA in GBM patient serum through biopsy, dysregulated lncRNAs may hold the potential to serve as biomarkers to offer a handy and cheap approach for GBM early diagnosis and prognosis. However, to our best knowledge, lncRNA has not been applied in GBM early diagnosis and prognosis at present, highlighting that there is an urgent need to identify more lncRNAs that could steady serve as biomarkers used in GBM clinical diagnosis and prognosis.

### 5.2 LncRNAs Used as Circulatory Biomarkers

**FIGURE 5:**
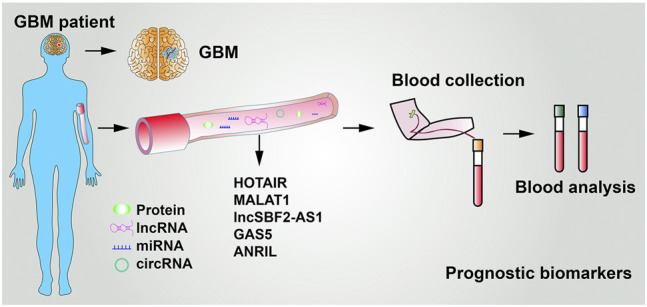
LncRNAs used in GBM patient diagnosis and prognosis.

To date, the diagnosis of GBM mainly relies on magnetic resonance imaging. In addition, compared with biopsy of focal tumor lesions, liquid biopsy is noninvasive, therefore, being an ideal diagnostic strategy. Several other tumor-associated lncRNAs can be detected in the body fluid (Huang et al., 2020a). As shown in Figure 5 and Table 2, five lncRNAs were detected to be highly expressed in the serum of GBM patients. For instance, Tan et al. (2018) reported that the serum levels of HOTAIR were markedly higher in GBM patients than in low-grade glioma, with a sensitivity of 86% and specificity of 88%. Cao et al. (2016) showed that the expression of MALAT1 was significantly increased in glioma specimens than in noncancerous brain tissues and MALAT1 expression markedly correlated with glioma grades. Importantly, serum levels of MALAT1 have also been applied in various types of cancer diagnosis and prognosis (Duan et al., 2016; Zhang et al., 2017c). Zhang et al. proposed that GBM cells remodel the tumor microenvironment by enhancing tumor TMZ-resistance through secreting the oncogenic lncSBF2-AS1–enriched exosomes. Therefore, exosomal lncSBF2-AS1 in human serum may act as a possible diagnostic marker for therapy-refractory GBM (Zhang et al., 2019). Clinically, high serum levels of lncSBF2-AS1 in exosomes were found to be correlated with worse responses to TMZ treatment in patients with GBM. Shen et al. also uncovered GAS5 expression levels in serum samples from 106 GBM patients. The results showed that high GAS5 expression in level is linked to a decreased likelihood of death, recurrence, and progression (Shen et al., 2018). Moreover, Sun et al. revealed that lncRNA-ANRIL expression levels were remarkably higher in GBM patient serum than in healthy people. The expression of lncRNA-ANRIL was inversely correlated with GBM patient clinical outcomes. Inhibition of lncRNA-ANRIL suppressed GBM cell invasion and avoided cellular apoptosis (Sun et al., 2021). All of these data suggest that serum levels of HOTAIR, MALAT1, lncSBF2-AS1, GAS5, lncRNA-ANRIL, and several unidentified lncRNAs have great potential to act as biomarkers utilized for glioma diagnosis and prognosis (Figure 5 and Table 2). However, it is worth mentioning that multiple alternative biomarkers have been proposed and investigated. Nevertheless, clinical trials and prospective validations are required before those can be regarded as clinically viable serum biomarkers for GBM.

**TABLE 2:** Serum lncRNAs used for diagnosis and prognosis of GBM patients.

Serum lncRNAs	Prognosis value	Reference
HOTAIR	Highly expressed in serum level and higher HOTAIR expression associated with worse survival of GBM patients	Tan et al. (2018)
MALAT1	Highly expressed in serum level and higher MALAT1 expression associated with TMZ resistance	Cao et al. (2016)
lncSBF2-AS1	Highly expressed in serum level	Zhang et al. (2019)
GAS5	Highly expressed in serum level and high GAS5 expression associated with recurrence and progression of GBM patients	Shen et al. (2018)
lncRNA-ANRIL	Highly expressed in serum level and higher ANRIL expression inversely associated with GBM patients’ prognosis	Sun et al. (2021)

### 5.3 Diagnostic Potentials of lncRNAs in GBM

As previously summarized in Table 1, a large number of lncRNAs, abnormally expressed in glioma as compared with a normal brain, can be used to distinguish glioma patients from healthy cohorts. However, some of those lncRNAs were also documented to be dysregulated in other cancers, such as lung cancer and liver cancer, leading to decreased reliability. Therefore, lncRNAs in combination with other biomarkers, especially the well-known glioma prognostic biomarker, isocitrate dehydrogenase (Hartmann et al., 2009), is more possible to be a favorably diagnostic biomarker instead of evaluating lncRNAs alone. The valuable significance of lncRNAs used in GBM diagnosis and prognosis goes by the fact that lncRNAs are characterized by tissue- and cell-specific expression in a variety of tumors. As previously mentioned, a vast majority of lncRNAs are deregulated in GBM compared with normal brain tissue, which is of clinical significance for the early diagnosis and prognosis of GBM patients. Wang et al. identified 1997 annotated ncRNAs by performing high-throughput microarray in 220 human glioma tissues and found that the expression of HOXA11-AS was dramatically increased in classical and mesenchymal subtype glioma than that in neural and proneural subtypes, indicating that HOXA11-AS holds great potential to act as a useful biomarker for classifying glioma subtypes (Wang et al., 2016a).

To date, surgical resection is the only likely curative strategy for low-grade glioma, while GBM in the late stage is largely incurable. Thus, new biomarkers and therapeutic targets are urgently required for advancing the diagnosis and clinical therapy of GBM. Ever since the discovery of lncRNA in fetal liver tissue in 1990 (Brannan et al., 1990b), aberrant lncRNA biogenesis has been reported to be observed in the pathogenesis of a variety of diseases, including GBM. In GBM, Mu et al. implemented high-throughput RNA sequencing in three fresh GBM patient specimens and three normal brain tissues from craniocerebral trauma patients and identified that 183 lncRNAs were significantly dysregulated in glioma (Mu et al., 2020). Among the highly expressed lncRNAs, BCYRN1 was recognized to be the most highly expressed in GBM, and further functional studies demonstrated that BCYRN1 significantly promotes GBM cell proliferation and migration (Mu et al., 2020). Li et al. characterized 247 immune-correlated lncRNAs from 529 low-grade glioma samples and five nontumor brain tissue samples in TCGA public database. Cox regression analysis demonstrated that 16 immune-related lncRNA expressions are correlated with the prognosis in low-grade glioma patients (Li and Meng, 2019). As shown in Table 1, we summarized the dysregulated lncRNAs in glioma and their unique biological significance in glioma occurrence and malignant development. Given the relatively easier examination of lncRNA in GBM patient serum through biopsy, dysregulated lncRNAs may hold the potential to serve as biomarkers to offer a handy and cheap approach for GBM early diagnosis and prognosis. However, to our best knowledge, lncRNA has not been applied in GBM early diagnosis and prognosis at present, highlighting that there is an urgent need to identify more lncRNAs that could steadily serve as biomarkers used in GBM clinical diagnosis and prognosis.

### 5.4 Therapeutic Potentials of lncRNAs in GBM

At present, the prognosis of GBM is extremely poor, partially owing to the lack of a therapeutic target. Recently, many studies demonstrated that novel therapeutics targeting lncRNAs are fairly effective in inducing suppression in a variety of tumor types. For example, Liu et al. (2020d) revealed that inhibiting lncRNA AGPG using antisense oligonucleotides (ASO) significantly restrained tumor growth in lung patient–derived xenograft (PDX) models. Xiu et al. (2019) recognized that ASO specifically targets LINC02273 dramatically repressed breast cancer invasion and metastasis *in vivo*. Han et al. (2020c) reported that targeting circLONP2 using ASO substantially inhibits colorectal carcinoma cell invasive ability. These studies revealed that targeting lncRNAs is a new strategy to promote the next generation of cancer therapeutics to advance cancer treatment. However, few efforts have been devoted to the application of ASO-targeted lncRNAs in GBM therapeutic at present (Bennett, 2019; Scoles et al., 2019). The abnormal lncRNA expression level in the specimens of clinical glioma is associated with tumor grades and differentiation status, and both of them have valuable clinical significance in the diagnosis and sub-classification of glioma (Stupp et al., 2009) and prognostication (Rutenberg-Schoenberg et al., 2016; Slack and Chinnaiyan, 2019). Given that GBM is intracranial, the blood–brain barrier (BBB) consists of a specific barrier for drug delivery. The lack of relevant investigation might be attributed to the fact that it is difficult to cross the BBB for lncRNAs. Therefore, chemical modifications of ASO that enable them to counteract the BBB are urgently required for advancing GBM therapy and are of paramount clinical significance to overcome this deadly disease.

A variety of lncRNAs have been documented to play critical roles in the initiation and progression of GBM, which can be utilized as targets for targeted therapy for GBM patients. At present, emerging strategies, including ASOs, locked nucleic acids (LNAs), peptide nucleic acids (PNAs), and morpholino oligonucleotides, have been applied in targeting lncRNAs (Liu et al., 2018; Shahzad et al., 2021). ASOs are single-stranded oligonucleotides with complementary sequences to the target RNAs and repress target RNA biological function by directly binding with partners, which can be used to target mRNAs, miRNAs, lncRNAs, circRNAs, and piRNAs and can be delivered naked without using vehicle delivery (Wang et al., 2019c; Petrescu et al., 2019). As shown in Table 3, ASOs have been documented to execute profound effects in suppressing MALAT1 expression and attenuate GBM cell metastasis and proliferation *in vivo* (Wan et al., 2014). Morpholinos (MO) are 25-nt nonionic DNA analogs that hybridize to the cognate site of target RNA and trigger target RNA degradation. As revealed by Lu et al., MO delivered *via* nanoparticles to target lncRNA DANCR in a human ovarian cancer xenograft model convincingly inducing strong repression of tumor growth. However, to the best of our knowledge, there are no studies on the MO used in targeting lncRNA, suggesting that there is an urgent need to apply those advancing strategies in target crucial lncRNAs promoted GBM progression to validate the clinical applications of these lncRNAs in GBM patients. As mentioned in Figure 2D, circRNA E-cadherin is overexpressed in GBM and promotes glioma stem cell tumorigenicity by encoding a small peptide, namely, C-E-Cad. Notably, the inhibition of C-E-Cad exhibits the dramatically antitumor activity *via* suppressing EGFR signaling in GBM (Gao et al., 2021).

**TABLE 3:** LncRNAs potentially used for targeted therapy for GBM patients.

Targeted lncRNAs	Strategy	Therapeutic effects	Reference
MALAT1	ASO	Suppressing GBM cell metastasis and proliferation	Wan et al. (2014)
circRNA E-cadherin	Monoclonal antibody	Suppressing GSC tumorigenicity	Gao et al. (2021)

Apart from directly targeting lncRNAs, regulating blood–tumor barrier (BTB) permeability is another way to target GBM. The BTB is a structure that comprises vascular endothelial cells, basement membrane, and glioma cells, which have the capacity to seriously impede the entry of drugs into the tumor microenvironment, leading to extremely unfavorable drug efficacy and worse patient prognosis (Letrado et al., 2018; Vogelbaum, 2018). Given that lncRNAs participate extensively in the malignant progression of GBM, the majority of these lncRNAs are uniquely and differentially expressed in GBM compared to their corresponding normal tissues (Li et al., 2018). Thus, characterizing compounds or other inhibitors target lncRNAs that influence BBB permeability to enhance chemotherapy and increase drug efficacy is one of the research directions for GBM-targeted therapy. Li et al. revealed that targeting HOTAIR enhances BTB permeability by decreasing miR-148b-3p, leading to a decrease in the expression of GBM-microvascular endothelial cell tight junction by targeting USF1 (Sa et al., 2017). Moreover, BTB permeability was shown to be augmented by silencing LINC00174 (Shi et al., 2019b), FBXL19-AS1 (Liu et al., 2020e), and lnc00462717 (Wang et al., 2017) in glioma tissue. Overcoming the obstacle of the BTB to increase the local concentration of chemotherapeutic agents in glioma and thereby increasing therapeutic efficacy is a promising strategy. Therefore, characterizing more appropriate therapeutic targets has been a major concern.

## Conclusion and Perspective

GBM is one of the most devastating diseases globally. Therefore, a better understanding of the molecular basis of the initiation and progression of GBM is of great significance to the development of new therapies for patients with GBM. LncRNAs have been well-established to play essential roles in gene regulation and multiple disease initiation and progression. Based on the current knowledge, significant progress has been achieved in characterizing lncRNAs and in deciphering their biological function and clinical potential in GBM occurrence and development over the previous decades. In addition, a variety of lncRNAs were found to function as indispensable factors in the tumorigenesis and progression of GBM. However, several fundamental questions remain to be addressed in the future. First, thousands of characterized lncRNAs have been documented to play key roles in the regulation of gene expression and execute diverse biological activities in a wide range of tumor types at present (Deveson et al., 2017; Hu et al., 2018b), and numerous lncRNAs have been reported to play integral roles in the initiation and development of GBM malignancy, including sustaining stemness, uncontrolled proliferation, invasion, abnormal angiogenesis, and resistance to conventional therapeutics (Peng et al., 2018b). However, among the annotated lncRNAs, only a small proportion of lncRNAs have been functionally recognized, and only a very small proportion of lncRNA-related studies shed new insights into lncRNA biological functions in the initiation and progression of GBM, such as encoding micro-peptides associated with GBM progression, alterations in the processes or structure of proteins, and precisely regulating the tumorigenesis, have not yet been fully understood in GBM pathogenesis (Gibb et al., 2011; Chen and Shen, 2020; Liu et al., 2020f; Yuan et al., 2020). Targeting those oncogenic drivers of GBM may achieve new progress of GBM clinical therapy. Thus, there is still a long way to translate basic scientific discoveries into clinical therapeutics for GBM patients. In addition, given that the dysregulation of lncRNAs and easy detection of lncRNAs through biopsy have great potential to provide novel insight into predictive and prognostic biomarkers for GBM, there is an urgent need to decode those dysregulated lncRNA functions and translate them into biomarkers for glioma patient diagnosis and prognosis.

Second, ceRNA regulatory networks were typically highlighted in the vast majority of GBM-related lncRNA studies, highlighting that it is important to illustrate the functional principles of lncRNAs in GBM. This implies that the current view of the functional role of lncRNAs *via* competition for miRNAs between RNAs is too simplistic and is essentially required to decode the complicated function by deciphering the other binding partner, such as protein or DNA. In addition, many dysregulated lncRNAs in GBM and the underlying molecular mechanisms still need to be further investigated. Given that lncRNAs primarily execute their functions through binding with other biomolecules, including DNA, RNA, and protein; the senior biological structures of the binding domains are of particular significance. We expect that further studies may be centered on uncovering lncRNA-binding motifs, which could generate more novel RNA-based targets in the development of new therapeutics for cancer treatment.

Third, at present, the therapeutic approaches based on lncRNA structures remain limited in application for the relatively poor stability of lncRNAs. Exosome delivery systems hold the potential to dramatically increase the bioavailability by preserving the integrity of macromolecules, and the engineering of ligand-dependent exosome membranes that specifically target brain tissues is also drawing great interest, leading to targeted therapy being more efficient and specific. It is worth noting that the BBB consists of a specific barrier for drug delivery. Therefore, it is of great significance to develop new drug delivery systems that embody lncRNAs or other compounds that have the capacity to cross the BBB for GBM clinical therapeutics.

Fourth, diverse cancer cells within the same tumors present dramatic genetical and phenotypical distinctions, namely, intratumor heterogeneity. Intratumor heterogeneity is considered an important contributor to glioma progression and therapeutic resistance (Gerlinger et al., 2012). So far, most studies of ceRNA regulation have been performed at a cell-population level. It remains to be elucidated how ceRNA crosstalk differs from cell to cell in a genetically identical or genetically heterogenous population. It also remains unknown how ceRNA regulatory networks rewire during tumor evolution and contribute to the therapeutic resistance mechanism of a cancer cell. By combining the powerful single-cell techniques (Dalerba et al., 2011; Ramsköld et al., 2012; Wang et al., 2016b), bioinformatics, and mathematical modeling approaches, we may be able to open a new avenue on how lncRNA crosstalk contributes to glioma heterogeneity, which can further boost the clinical implications of lncRNAs.

Herein, we summarize the functional roles of emerging lncRNAs and their underlying mechanisms in the initiation and progression of GBM. Finally, we describe the potential application of lncRNAs in GBM as diagnostic and prognostic biomarkers, as well as the potential therapeutic target of developing lncRNA-targeted therapy.

## Glossary

**GBM**: Glioblastoma
**TMZ**: Temozolomide
**ncRNAs**: Noncoding RNAs
**lincRNAs**: Intergenic lncRNAs
**circRNAs**: Circular Intronic RNAs
**ceRNA**: Competitive endogenous RNA
**RBP**: RNA-binding protein
**FOXM1-AS**: A long noncoding RNA antisense to FOXM1
**sORFs**: Open reading frames
**GSC**: Glioma stem cell
**MGMT**: O-6-methylguanine-DNA methyltransferase
**GECs**: Glioma-associated endothelial cells
**CXCR7**: Chemokine receptor 7b
**ASO**: Antisense oligonucleotides
**PDX**: Patient-derived xenograft
**BBB**: Blood–brain barrier
**LNAs**: Locked nucleic acids
**PNAs**: Peptide nucleic acids
**MO**: Morpholinos
**BTB**: Blood-tumor barrier
**XIST**: X inactive specific transcript
**SOX2**: SRY-box transcription factor 2
**HIF-1α**: Hypoxia-inducible factor 1
**PDIA3P1**: Protein disulfide isomerase family A member 3 Pseudogene 1
**TF**: Transcription factor
**SWINGN**: SWI/SNF interacting GAS6 enhancer noncoding RNA
**SMARCB1**: SWI/SNF-related, matrix-associated, actin-dependent regulator of chromatin, subfamily b, member 1
**HOTAIRM1**: HOXA transcript antisense RNA, myeloid-specific 1
**STAT1**: Signal transducer and activator of transcription 1
**RPL36**: Ribosomal protein
**SNHG4**: Small nucleolar RNA host gene 4
**SNHG15**: Small nucleolar RNA host gene 15
**c-Met**: MET proto-oncogene, receptor tyrosine kinase
**GAPLINC**: Gastric Adenocarcinoma–associated, positive CD44 regulator, long intergenic noncoding RNA
**MALAT1**: Metastasis-associated lung adenocarcinoma transcript 1
**ZHX1**: Zinc fingers and homeoboxes 1
**BCYRN1**: Brain cytoplasmic RNA 1
**CUEDC2**: CUE domain containing 2
**PTEN**: Phosphatase and tensin homolog
**ADAMTS9-AS2**: ADAM metallopeptidase with thrombospondin type 1 motif 9 antisense RNA 2
**AKT1**: AKT serine/threonine kinase 1
**TUG1**: Taurine upregulated 1
**Notch1**: Notch receptor 1
**MATN1-AS1**: MATN1 antisense RNA 1: MATN1 antisense RNA 1
**E2F6**: E2F transcription factor 6
**p21**: Cyclin-dependent kinase inhibitor 1A
**DLGAP1-AS2**: DLGAP1 antisense RNA 2
**EMT**: Epithelial-mesenchymal transition
**YAP1**: Yes1-associated transcriptional regulator
**ST7-AS1**: ST7 antisense RNA 1
**ZNF596**: Zinc finger protein 596
**EZH2**: Enhancer of Zeste 2 polycomb repressive complex 2 subunit
**STAT3**: Signal transducer and activator of transcription 3
**ALKBH5**: AlkB homolog 5
**FOXM1**: Forkhead box M1
**EGFR**: Epidermal growth factor receptor
**LINC-PINT**: Long intergenic non-protein coding RNA, p53 induced transcript
**PAF1**: Paf1/RNA polymerase II complex component 1
**ZEB1**: Zinc finger E-box–binding homeobox 1
**HMGB1**: High mobility group box 1
**HOTAIR**: HOX transcript antisense RNA: HOX transcript antisense RNA
**NNT-AS1**: NNT antisense RNA 1
**PRMT1**: Protein arginine methyltransferase 1
**ECONEXIN**: long intergenic nonprotein coding RNA 461
**TOP2A**: DNA topoisomerase II alpha
**MATN1-AS1**: MATN1 antisense RNA 1: MATN1 antisense RNA 1
**ROR1-AS1**: ROR1 antisense RNA 1: ROR1 antisense RNA 1
**LEF1-AS1**: LEF1 antisense RNA 1
**lncRNA-ATB**: lncRNA activated by TGF-beta
**BMP4**: Bone morphogenetic protein 4
**NEAT1**: Nuclear paraspeckle assembly transcript 1
**PAXIP1AS1**: PAXIP1 divergent transcript
**KLF14**: Kruppel-like factor 14
**Sox2OT**: SOX2 overlapping transcript
**RCC**: Renal cell carcinoma
**HDACI**: Histone deacetylase inhibitors
**DNMT**: DNA methyltransferase
**BET**: Bromodomain and extraterminal
**PCGEM1**: Prostate-specific transcript
**HNAR**: Ribosomal protein L13a pseudogene 20
**ZFPM2-AS1**: ZFPM2 antisense RNA 1
**NFIA-AS2**: NFIA antisense RNA 2
**GAS5**: Growth arrest–specific 5: Growth arrest–specific 5
HOTAIR: HOX transcript antisense RNA: HOX transcript antisense RNA
**DC**: Dendritic cells
**KCNQ1OT1**: KCNQ1 opposite strand/antisense transcript 1
**AHIF**: Antisense transcript of hypoxia-inducible factor-1α
**SP1**: Sp1 transcription factor
**CASC2**: Cancer susceptibility 2
**TP73-AS1**: TP73 antisense RNA 1
**CDK6**: Cyclin-dependent kinase 6
**H19**: H19 imprinted maternally expressed transcript
**VASH2**: Vasohibin 2
**HULC**: Hepatocellular carcinoma upregulated long noncoding RNA
**PI3K**: PI3K phosphatidylinositol 3-kinase
**mTOR**: Mechanistic target of rapamycin kinase
**NKILA**: NF-κB-interacting long noncoding RNA
**ZNF655**: Zinc finger protein 655
**USP44**: Ubiquitin-specific peptidase 44
**PD-L1**: Programmed cell death 1 ligand 1
**TIM-3**: Hepatitis A virus cellular receptor 2
**ROR1-AS1**: ROR1 antisense RNA 1: ROR1 antisense RNA 1
**TCGA**: The Cancer Genome Atlas
**HOXA11-AS**: HOXA11 antisense RNA
**CCND2-AS1**: CCND2 antisense RNA 1
**GAS5**: Growth arrest–specific 5: Growth arrest–specific 5
**ANRIL**: CDKN2B antisense RNA 1
**DANCR**: Differentiation antagonizing nonprotein coding RNA
**FBXL19-AS1**: FBXL19 antisense RNA 1
